# Comparative study of extrapolative factors linked with oxidative injury and anti-inflammatory status in chronic kidney disease patients experiencing cardiovascular distress

**DOI:** 10.1371/journal.pone.0171561

**Published:** 2017-02-08

**Authors:** Mahmood Rasool, Muhammad Abdul Basit Ashraf, Arif Malik, Sulayman Waquar, Shahida Aziz Khan, Mahmood Husain Qazi, Waseem Ahmad, Muhammad Asif, Sami Ullah Khan, Ahmad Zaheer, Muther Mansoor Qaisrani, Abdul Rehman Khan, Aamir Iqbal, Amir Raza, Saima Iram, Kashif Kamran, Asim Iqbal, Mohammad Zahid Mustafa, Hani Choudhry, Mazin A. Zamzami, Wesam H. Abdulaal, Mohammad Sarwar Jamal

**Affiliations:** 1 Center of Excellence in Genomic Medicine Research (CEGMR), King Abdulaziz University, Jeddah, Saudi Arabia; 2 Institute of Molecular Biology and Biotechnology (IMBB), The University of Lahore, Lahore, Pakistan; 3 King Fahd Medical Research Center, King Abdulaziz University, Jeddah, Saudi Arabia; 4 Center for Research in Molecular Medicine (CRiMM), The University of Lahore, Lahore, Pakistan; 5 Department of Biotechnology and Informatics, BUITEMS, Quetta, Pakistan; 6 Department of Botany, Women University of Azad Jammu & Kashmir, Bagh, Pakistan; 7 National Institute for Biotechnology and Genetic Engineering (NIBGE), Faisalabad, Pakistan; 8 Department of Botany, Ghazi University, Dera Ghazi Khan, Pakistan; 9 Obesity & Diabetes Research Laboratory, Department of Chemistry, University of Azad Jammu & Kashmir, Muzaffarabad, Pakistan; 10 Bolan Medical Hospital, Quetta, Pakistan; 11 Faculty of Life Sciences, University of Balochistan, Quetta, Pakistan; 12 CASVAB, University of Balochistan, Quetta, Pakistan; 13 Department of Biochemistry, Cancer Metabolism and Epigenetic Unit, Faculty of Science, Center of Innovation in Personalized Medicine, Cancer and Mutagenesis Unit, King Fahd Center for Medical Research, King Abdulaziz University, Jeddah, Saudi Arabia; 14 Department of Biochemistry, Cancer Metabolism and Epigenetic Unit, Faculty of Science, Cancer and Mutagenesis Unit, King Fahd Center for Medical Research, King Abdulaziz University, Jeddah, Saudi Arabia; Rosalind Franklin University of Medicine and Science, UNITED STATES

## Abstract

**Background:**

Chronic kidney disease (CKD) is a group of heterogeneous abnormalities affecting the function and structure of the kidney and mostly further proceeds to cardiovascular damage prior to end stage renal disease (ESRD). The oxidative insult and inflammatory mediators have some undefined role in CKD and cardiovascular complications. It is therefore, aimed at to pin point the predictive factors in the development of cardiovascular disorder in patients with chronic kidney disease.

**Methods:**

Fifty patients of CKD experiencing cardiovascular distress and twenty normal individuals having same age and sex acted as control during these observations. Blood samples (Each 5 ml) were drawn and subjected to centrifugation for 10–15 minutes to separate the serum at 4000-5000rpm. The levels of MDA, GSH, SOD, CAT, VIT C, VIT E, IL-1, TNF-alpha, nitric oxide (NO) and advanced oxidation protein products (AOPPs) were estimated and analyzed.

**Results:**

The nitric oxide levels in the CKD patients decreased significantly (13.26±1.25 ng/ml) compared to controls (42.15±5.26 ng/ml). The serum vitamin E and C levels in these patients recorded 2.15±0.25 μg/ml and 0.97±0.09 μg/ml respectively as against their assigned controls which read 6.35±1.22 μg/ml and 3.29±0.25 μg/ml. Furthermore, a significantly higher level of Malondialdehyde **(**MDA) as1.25±0.07 nmol/ml was observed in CKD patients viz-a-viz relevant control. However, the serum SOD, catalase (CAT) and GSH levels in the same patients registered a significant decline as evident from respective figures 0.07±0.002 μg/dl, 1.22±0.012 μmol/mol, and 3.25±1.05 μg/dl. The control for these was observed as0.99±0.06 μg/dl, 3.19±0.05 μmol/mol, and 8.64±0.03 μg/dL. On the other hand, the IL-1 levels in the CKD patients found quite higher (402.5±18.26 pg/ml). This clearly points to substantial increase in oxidative insult and reduced NO levels leading to the renal and cardiovascular damage.

**Conclusion:**

Observations support the fact that the decrease in anti-oxidative capacity accompanied by higher inflammatory mediators in CKD is indicative of oxidative stress, consequently leading to CKD progression, in all probability to cardiovascular insult. The outcome reiterates that strategies be designed afresh to contain CKD progression to cardiovascular complications and ESRD. One way could be to focus on early detection of stress related to the disease. It requires analyzing the factors related to stress, such as the one reported here. Linking these factors with the symptoms could be a crucial step forward. And further, the disease could be monitored in a more disciplined manner.

## Introduction

CKD (Chronic kidney disease) is group of heterogeneous conditions affecting the structure and function of kidney. Acute kidney injury, intake of nephrotoxins, weight gain, smoking and increasing age are the factors linked to it [1]. Structural features attached like, augmentation in glomerulosclerosis, renal vasculopathy, tubular inflammation, fibrosis, atrophy together with presence of scar tissue, have also been thoroughly identified [2]. Earlier pathological variations in kidney may occasionally happen in the absence of clinical presentation due to high adaptability [3]. Notwithstanding, these physiological studies, what is more serious is CKD progression once adaptive threshold is broken, progression to end stage renal disease is impending. Hence, identifying its stage is imperative. For that, determining the loss of glomerular filtration rate (GFR) is the primary clinical manifestation. The CKD acquire more serious proportion as patients are more prone to die of cardiovascular diseases (CVD) than the actual renal failure.

The association between CKD and CVD assumes greater proportion since both have same risk factors and the disease related circumstances in one system can adversely affect the other system. Understandly, in the prior CKD stage, the incidence of CVD events becomes higher could possibly due to glomerular filtration rate (GFR), aggravate the severity of CVD abnormalities. This is reinstated by a study, where in CKD patients with stage III and IV, the incidence of CVD is 4–5 times higher than population [4]. Hypertension is a highly implicated etiological factor also for the induction of CVD in CKD patients. It is because; sodium retention and activation of renin angiotensin system (RAS), are the underlying mechanisms get stressed. Also directly, hypertension causes cardiac deterioration in CKD, as the induction of left ventricular hypertrophy takes place. And further consequently results in the decrease of coronary reserves and capillary density, leading to coronary ischemia [5]. In CKD further, uremia results in production of cyanate in the presence of hydrogen peroxide (H_2_O_2_) and myeloperoxidase (MPO) mediates carbamylated protein synthesis that combines with high density lipoprotein (HDL). On its part, carbamylated HDL reduces lecithin cholesterol acyl transferase (LCAT) activity, so important in cholesterol esterification and HDL maturation and incidently, leading to CVD events in CKD patients [6].

The CKD persons are linked to the complications in cellular respiration as well–directly or indirectly to the stress. Oxidative stress occurs either due to inability of scavenging free radicals by antioxidative enzymes or excessive reactive oxygen species (ROS) production by electron transport chain (ETC) in mitochondria as a primary source [7]. It is indicated by compromised mitochondrial respiration in CKD patients, resulting in diminished ATP production affecting a reduction in glutathione mediated redox balance because the glutathione formation rate is ATP dependent [8,9]. Hence, the alternate ways to reduce such complications are vital. The antioxidative defense system is one such mechanism, if primary antioxidants; superoxide dismutase, catalase and glutathione peroxidase are impaired [10]. Their decrease directly retard processing of superoxide free radical (O^.^_2_¯) as its level is increased; cell membrane is damaged which is determined on its part by malondialdehyde (MDA) levels [11] and protein oxidation assessed by advanced oxidation protein products (AOPPs) [10].

There is another angle to impairement of respiratory process involving enzyme dealing NADPH–another energy carrier molecule. Tumor necrosis factor-alpha (TNF-α), interleukin 1 and 6 induce NADPH oxidase for generation of superoxide free radicals [12]. Thus, there is dual increase in free radicals; one by reduced antioxidative capacity and two by inflammatory mediators. There are reports for the formation of peroxynitrite (ONOO¯) radicals by the reaction of O^.^_2_¯ free radicals to nitric oxide (NO) mediated by nitric oxide synthase (NOS) inducing lipid peroxidation further [13]. The NO utilization, results in vasoconstriction causing vascular injury.

In CKD, vitamins C and E (α-tocopherol) have a pivotal significance. There are non-enzymatic antioxidants. The synthesis of active form of vitamin C, ascorbate is dependent on reduced glutathione which is reduced in CKD [14]. Vitamin E hampers lipid peroxidation and is restored by vitamin C thus both are inter-dependent [15]. This is a significant example of an antioxidant network susceptible to malfunctioning in CKD patients. Hence, the main aim of this study is to investigate the implications of extrapolative factors useful in these diseases especially in development of cardiovascular disease in such patients also diagnosed with chronic kidney impairements causing renal failure.

## Materials and methods

Fifty patients affected with chronic kidney diseases were enrolled in the study at Jinnah Hospital, Lahore. Clinical diagnosis and history of the patients were obtained from hospital medical records. Concurrently, twenty healthy people acted as control. Informed written consent was obtained prior to the start of study as per Helsinki declaration. The Study was approved by the ethical committee of the University of Lahore. Five ml of blood was drawn from each patient and control subjects and centrifuged at 4000 rpm for serum separation and the samples transported immediately to the laboratory for further processing. All the chemical and reagents used in this study were of analytical grades and purchased from Sigma chemicals Co. (St. Louis, Mo, USA).

### Inclusion and exclusion criteria

All the patients included were at the stage V of CKD with confirmed clinical reports of glomerular filtration rate (GFR) of less than 15. The CKD patients with the history of hyperextension, cigarette smoking, and alcohol consumption were excluded from the study.

### Estimation of Glutathione (GSH)

Glutathione was estimated as per protocol of Moron *et al*., [16]. The 100μl of serum was taken and added 0.02M (2.4μl) EDTA and ice-cooled (10 min), Followed by addition of 2 ml distilled water. To this 50.0μl of TCA (50%) was added and incubated on ice (10–15 min). Samples were then centrifuged (3500 rpm). The supernatant was removed and added with 2ml of 0.15M Tris. HCl and 0.05ml (DTNB). Absorbance was measured at 412nm.

### Estimation of Catalase (CAT)

Catalase (CAT) was determined by the method of Aebi, [17]. 100μl of sample was added in the tube followed by 1.9ml of phosphate buffer and 1ml of H_2_O_2_. At last three absorbance were measured after every minute at the wavelength of 240nm.

### Determination of Superoxide Dismutase (SOD)

Superoxide dismutase (SOD) was estimated as perKakkar procedure [18]. The 100μl of sample was taken in tube, added with 1.2ml of PBS, 100μl of phenazine methosulfate, 300μl of NBT and 200μl of NADH. Thereafter, 100μl of Glacial acetic acid and 4ml of 2-propanol was added further in tube and centrifuged (3000 rpm, 10 min). The absorbance was taken at 560nm.

### Estimation of Malondialdehyde (MDA)

This method used Ohkawa *et al* Procedure [19]. 200μl of the sample was taken in the tube,to which 200μl of 8.1% SDS and 1.5ml of 20% acetic acid was added. Later 1.5ml of 0.8% TBA and 600μl of distilled water along with 4ml of 2-propanol was supplemented. It was centrifuged (4000 rpm, 10 min) and supernatant was removed for measuring absorbance at 532nm using UV-1100 spectrophotometer.

### Advanced Oxidative Protein Products (AOPPS) determination

AOPPs were estimated by the method of Witko-Sarsat *et al*., [20]. 200μl of sample was first diluted with PBS, then 10μl of KI (1.16M) and 20μl of acetic acid was added. The sample centrifuged (5000 rpm, 5min) and absorbance was taken at 340nm on UV-spectrophotometer.

### Estimation of Nitric Oxide (NO)

Bories and Bories method employed for this purpose [21]. 100μl of Griess Reagent was added with 300μl of sample and supplemented with 2.6ml of distilled water followed by incubation (30 min). The absorbance was measured at 548nm.

### Estimation of vitamin C

For estimation of Vitamin C method of Chinoy *et al*., followed [22]. 100μl of the sample was added with 400μl of TCA 5% and centrifuged (3000 rpm, 10 min). 320μl of supernatant was separated and added with 130μl of DTC and allowed to heat (90°, 1hr). It was later ice-cooled and added with 600μl of sulphuric acid then subjected to absorbance at 520nm.

### Estimation of vitmain E

Vitamin E was estimated by the method of Rosenberg [23]. 200μl of the sample was supplemented with 200μl of ethanol, 200μl of n-hexane and distilled water. It was then centrifuged (3000 rpm, 10 min) and added with 25μl of Bathophenanlhroline, 75μl of ferric chloride and 50μl of Orthophosphoric Acid. Its absorbance was taken at 536nm.

### IL-1 and TNF-alpha determination

Estimation of interleukin-1 and tumor necrosis factor (TNF-alpha) were performed by using ELIZA kits (R&D Systems, MN, USA and Affimatrix) respectively).

### Statistical analysis

The Statistical Analysis was performed by SPSS version 17.0. Independent T-test was performed by taking average means of group populations. Pearson's correlation coefficient was used to assess correlations among different variables in CKD patients. The p-values were computed using one way ANOVA.

## Results

**Fig 1:**
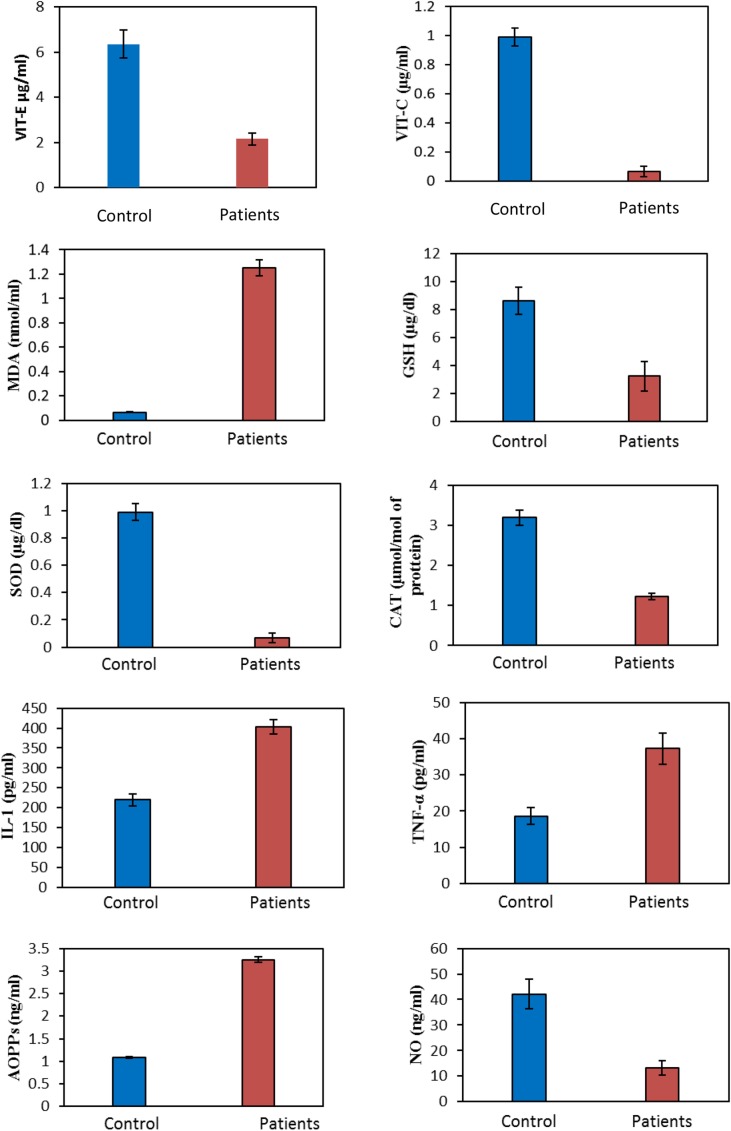
Biochemical profile of CKD patients experiencing CVD linked with oxidative injury and anti-inflammatory status.

The data presented in the Fig 1 shows the clear picture of different parameters estimated in the patients suffering from CKD. When oxidative stress biomarkers were estimated, increase in MDA levels was observed in the CKD patients as compared to controls (1.25±0.07 vs 0.07±0.01 nmol/ml). GSH levels in the CKD patients were markedly lower (3.25±1.05μg/dL) as compared to control (8.64±0.03 μg/dL). The serum SOD levels in the CKD patients lowered (0.07±0.002 μg/dL) in comparison to control group (0.99±0.06 μg/dL). The catalase (CAT) levels in CKD patients lowered in comparison to the control group (1.22±0.012 vs 3.19±0.05 μmol/mol of protein). When the levels of vitamins were measured, serum vitamin E levels in the CKD patients were 2.15±0.25 μg/ml while, in healthy individuals were 6.35±1.22 μg/ml. Likewise, the level of vitamin C in CKD patients was significantly (*p* = 0.021) low (0.97±0.09 μg/ml) as compare to controls (3.29±0.25 μg/ml). The IL-1 levels in the CKD patients were estimated as 402.5±18.26 pg/ml while in the healthy individuals as 219.65±15.26 pg/ml. The TNF-α levels in CKD were 37.26±4.26 pg/ml while in control ones 18.65±2.25 pg/ml. When the AOPPs levels were measured in CKD patients, the value was significantly higher (3.25±0.07 ng/ml) than those measured in controls (1.09±0.02 ng/ml). The nitric oxide levels in the CKD patients were significantly decreased as compared to those in the controls (13.26±1.25 vs 42.15±5.26 ng/ml). The significant findings were that reduction in NO and vitamin C results in increase in IL-1 which mediates excessive ROS generation resulting in cardiovascular insult in CKD patients prior to ESRD (Table 1).

**Table 1:** Result of all the variables among CKD patients and control.

Variables	Control (n = 20)	Patients (n = 50)	*p*-value
Vitamin E (μg/ml)	6.35±1.22	2.15±0.25	0.026*
Vitamin C(μg/ml)	3.29±0.25	0.97±0.09	0.021*
MDA (nmol/ml)	0.07±0.01	1.25±0.07	0.046*
GSH (μg/dL)	8.64±0.03	3.25±1.05	0.033*
SOD (μg/dL)	0.99±0.06	0.07±0.002	0.011*
CAT (μmol/mol of protein)	3.19±0.05	1.22±0.012	0.035*
IL-1 pg/ml	219.65±15.26	402.5±18.26	0.002*
TNF-α (pg/ml)	18.65±2.25	37.26±4.26	0.002*
AOPPs	1.09±0.02	3.25±0.07	0.032*
Nitric Oxide (NO)	42.15±5.26	13.26±1.25	0.023*

*Significant (p-value <0.05).

## Discussion

For the purpose to evaluate the relationship of oxidative stress, and pro-inflammatory cytokine status in CKD patients which have susceptibility to progress towards cardiovascular insult; and the correlations of ROS, antioxidants and cytokines were developed to attain the interrelationship among stress markers and antioxidants; like MDA, SOD, CAT, GSH, AOPPs, vitamin E and C were estimated. Moreover, their conclusive effects on NO, TNF-alpha and specifically IL-1 were also determined. Compromised antioxidant mechanisms are the early and progressive phenomenon in the advancement of CKD. Its progression results in atherosclerotic changes prior to ESRD in which the main role is of disturbance in redox balance. A recent report has shown that superoxide dismutase I (SODI) has a significant importance in maintaining redox balance in progressive kidney injury [24]. It can be impaired in CKD [25] leading to increase in superoxide free radicals generation thereby resulting in cellular apoptosis, a process central to structural and functional loss of renal tissue and endothelial dysfunction [26]. Catalase has a prominent role in hydrogen peroxide (H_2_O_2_) reduction, which plays a key role in anti-oxidative shield in kidney. The loss of its buffering activity results in oxidative stress and more vigorous renal fibrosis leads to progressive renal injury [27]. However, the role of catalase activity is still controversial in the CKD [28].

**Fig 2:**
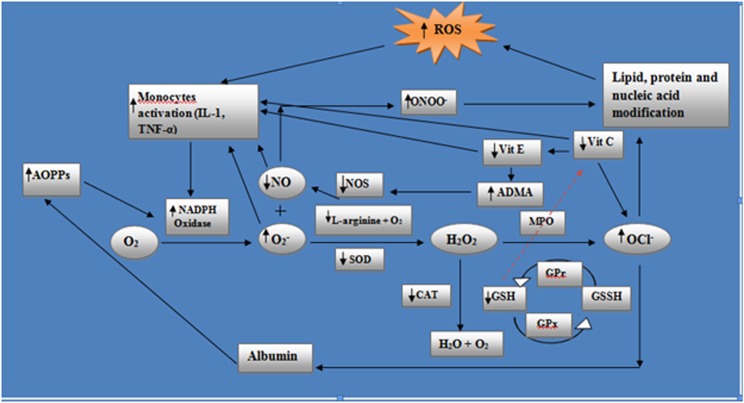
Oxidative injury is enhanced by inflammatory mediators (interleukins and TNF-α) as it provokes the activity of NADPH oxidase. The resultant superoxide anion (O_2_¯) not only in turn further activate monocytes to release inflammatory mediators but also react with nitric oxide (NO) to form peroxynitrite (ONOO¯) which induce lipid peroxidation and NO consumption, important in proper vascular functioning and this decrease in NO increases mainly IL-1. It generates free radicals which again stimulate monocytes. O_2_¯ processing into hydrogen peroxide (H_2_O_2_) is decreased as it is mediated by superoxide dismutase (SOD) which is deficient in CKD. H_2_O_2_ can be converted into hypochlorite ion (OCl¯) by myeloperoxidase (MPO) and into water and oxygen by catalase which may be decreased in CKD. Reduced glutathione (GSH) not only decreases H_2_O_2_ processing but also reduces vitamin C which has three effects, firstly it increases inflammatory mediators, secondly raises OCl¯ and thirdly limits vitamin E (vit E) recycling. Raised OCl¯ free radicals attack albumin to produce advanced oxidative protein products (AOPPs) which promote NADPH oxidase activity thus aggravating oxidative insult. Reduction in vitamin E results not only in increased inflammatory mediators but also in raised asymmetrical dimethylarginine (ADMA) levels which competes L-arginine (also decreased in CKD) for nitric oxide synthase (NOS) thus reduces its levels and hampers NO formation leading to cardiovascular injury.

Oxidative stress resulting in mitochondrial dysfunction induces ATP depletion as well as mitochondrial membrane potential loss. Depleted ATP synthesis causes reduction in glutathione levels as its rate of formation is ATP mediated, occurring in all segments of nephron except proximal tubule [29]. Effect of oxidative stress in the progression of disease is expressed in the Fig 2. Thus the mitochondrial membrane potential loss results in increase in mitochondrial permeability which in turn releases cytochrome C (CytC), a pro-apoptotic factor. Oxidative stress results in CytC dissociation from cardiolipin and forms an apoptosome with apoptotic peptidase activating factor- I, leading to activation of caspases causing apoptosis [30]. NADPH oxidase causes formation of superoxide free radical (O_2_¯) from molecular oxygen (O_2_) and this process is abnormally increased in phagocytic cells in the CKD [31]. There is a two way increase in O_2_¯, one by decrease in processing and other by increase in production. In the CKD, increased NADPH oxidase activity causes increase in carotid intima media thickness (IMT), can be taken as an indicator of coronary atherosclerotic events [32].

Peroxynitrite free radicals are formed when superoxide anion reacts with nitric oxide (NO), a potent vasodilator which induces nitrosative modification of lipids, proteins and nucleic acid [33]. L-arginine combines with oxygen in the presence of NOS to form NO. In the CKD patients, L-arginine production from kidneys is reduced as there is reduction in renal tissue mass [7]. Thus there is a dual decrease in NO levels, one by increase in its utilization in the form of ONOO¯ and the other by reduction in its synthesis. This nitric oxide is important in maintaining normal endothelial cell functioning and its decrease results in CVD [34]. Nitric oxide also enhances glomerular filtration rate (GFR), renal blood flow, natriuresis and diuresis, but its reduction leads to compromised renal performance [35]. Nitric oxide is also an inhibitor of leukocyte activation, its adhesion to endothelium by reducing cell adhesion molecules (CAM’s) and liberation of cytotoxic vasoconstrictor products like leukotrienes (IL-1), indicator of tubule interstitial fibrosis [36], (IL-6) biomarker of mesangial glomerulopathy [37] and cytokines (TNF-alpha) [38]. The present findings indicate, NO is inversely correlated with IL-1 (NO vs IL-1, r = -0.716**) (Table 2).These products enhance NADPH activity resulting in O_2¯_ generation which may stimulate cytokines production by activating NF-ҡB and activator protein-1 (AP-1) leading to overproduction of ROS [12]. Asymmetrical dimethylarginine (ADMA), a potent marker of cardiovascular outcome [39] is metabolized by dimethylarginine dimethylaminohydrolase (DDAH) which is sensitive to oxidative stress [40]. ADMA hampers NOS activity by competing with L-arginine thereby resulting in endothelial dysfunction [41]. In the CKD, impaired antioxidative defense participate in oxidative stress which may induce ADMA mediated processes that may expedite cardiovascular damage.

**Table 2:** Pearson’s correlation coefficients of different variables in CKD associated with CVD.

Variables	Vit-E	Vit-C	MDA	GSH	SOD	CAT	IL-1	TNF-α	AOPPs	NO
**VIT-E**	1	0.876**	-0.213	0.786**	-0.164	0.677**	0.692**	-0.677**	-0.193	-0.636**
		0.0034	.0117	0.0091	0.073	0.0481	0.0056	0.0030	0.0791	0.007
**VIT-C**		1	-0.465*	-0.745**	-0.191	-0.589**	-0.706**	0.705**	0.161	0-.597*
			0.0158	0.0019	0.1811	0.0018	0.0041	0.0965	0.0531	0.0155
**MDA**			1	.359*	0.145	0.311*	-0.498*	0.527*	0.151	0.458*
				0.0125	0.265	0.0316	0.0330	0.0132	0.560	0.015
**GSH**				1	0.164	0.854**	-0.768**	0.811**	0.231	0.813**
					0.156	0.0017	0.0026	0.0015	0.235	0.0018
**SOD**					1	0.189	-0.183	0.224	0.050	0.192
						0.235	0.192	0.326	0.166	0.326
**CAT**						1	-0.739**	0.807**	0.254	0.862**
							0.0043	0.0017	0.0625	0.0029
**IL-1**							1	-0.769**	-0.252	-0.716**
								0.0019	0.265	0.0027
**TNF-α**								1	0.222	0.840**
									0.329	0.0019
**AOPPs**									1	0.295
										0.229
**NO**										1

The * signifies single 0 after the decimal point, and ** tends to explain two zeros after decimal point and so highly significant.

Another marker of presence of oxidative stress is oxidative dependent protein damage which can be assessed by measuring advanced oxidative protein products (AOPPs). Serum albumin is the primary source of AOPPs when it is attacked by hypochlorous acid free radicals [42]. In the CKD patients, it is not only associated with renal failure progression but also very significantly have a relationship with cardiovascular events [43]. AOPPs activate NADPH oxidase and have an ability to trigger oxidative spurt of monocytes and neutrophils so may be involved in the inflammatory processes [44]. AOPPs levels were inversely correlated with GFR in the CKD patients [10].

Another parameter of oxidative upset is lipid peroxidation of cell membrane resulting in production of malondialdehyde (MDA) and its levels are increased in the CKD patients as renal tissue mass decreases due to cell damage [11]. Oxidative stress also induces low density lipoprotein (LDL) oxidation. So, LDL receptors cannot respond to oxidized low density lipoprotein (ox-LDL) which is taken by macrophages resulting in the synthesis of foam cells and atherosclerotic plaques [45].

As for the role of vitamins; vitamin C is an active water soluble antioxidant against lipid peroxidation. It also exhibits hypochlorous acid scavenging property to reduce its devastating effects [46]. Its deficiency in the CKD patients may be due to glutathione (GSH) deficiency as its recycling is GSH mediated [14], we noticed an inverse correlation between GSH and vitamin C (GSH vs Vit.C, r = -0.745**) and deduced that vitamin C deficiency may be due to reduced dietary intake. In view of some studies, vitamin C intake cannot alter oxidative stress and inflammatory mediators but according to our results vitamin C is inversely correlated with IL-1 (Vit.C Vs IL-1, r = -0.706**) and directly correlated with TNF-α (Vit.C Vs TNF-α, r = 0.705**) and reiterate that there is an inverse relationship between IL-1 and TNF-α (IL-1 Vs TNF-α, r = -0.769**). It is also capable to reduce vitamin E to its active form as its activity is dependent on vitamin C (Vit. C Vs Vit. E = 0.876**) and further suggests that this dependence might be deranged in the CKD [15,47,48]. Vitamin E (α-tocopherol), a potent lipid solvable antioxidant scavenges free radicals especially nitrosative and peroxyl radical, redily hamper lipid peroxidation [49,50]. It inhibits protein kinase C mediated NADPH oxidase activity leading to limit oxidative perturbance [51]. It is proposed that vitamin E is positively correlated with GFR as it keeps glomerular basement membrane integrity [49]. Vitamin E also impedes pro-atherogenic events like monocyte O_2_¯ discharge, IL-I β and TNF-α synthesis, neutrophil chemotaxis and their adhesion to the endothelium [52]. Our results indicate that vitamin E is negatively correlated with TNF-α (Vit.E Vs TNF-α, r = -0.677**) while positively correlated with IL-1 (Vit.E Vs IL-1, r = 0.692**) which are in contrast to relationship of vitamin C with the inflammatory mediators. It also reduces ADMA levels as it boosts DDAH activity involved in metabolism of ADMA thus, preserving NOS activity which results in regular cardiovascular functioning but its reduction results in decreased NO production [53]. Contrary to it, it has been reported that there is no relationship between ADMA and vitamin E levels [54]. In the CKD, decreased vitamin E levels result not only in aggravation of oxidative free radicals, inflammatory biomarkers but also reduction of vasodilator agents resulting in progression to cardiovascular damage and ESRD.

## Conclusion

The present study revealed the substantial decrease in anti-oxidative capacity accompanied by higher inflammatory mediators in CKD induces oxidative stress. The free radicals lead to nitrosative and chlorinative compounds which promote lipid and protein peroxidation respectively. This further propagates oxidative stress resulting in CKD progression and cardiovascular impairment. Reduction in nitric oxide results in raised IL-1 resulting inflammation and vascular injury. Independent observation shows a decrease in vitamin E which as established earlier promotes lipid peroxidation by increasing NADPH activity and inflammatory mediators. Since, vitamin C resynthesizes vitamin E and its formation is regulated by glutathione, already deficient in CKD. Thus strategies could be developed using present observations to limit CKD progression to cardiovascular injury and ESRD and for this, focus on early detection and slowing disease progression is of prime importance. In this regard, oxidative stress and inflammatory mediators profile must be investigated at an early stage of disease to control devastating effects.
